# Impact of Electrical Stimulation on Mental Stress, Depression, and Anxiety: A Systematic Review

**DOI:** 10.3390/s25072133

**Published:** 2025-03-28

**Authors:** Sandra Mary Prasad, M. N. Afzal Khan, Usman Tariq, Hasan Al-Nashash

**Affiliations:** 1 Bioscience and Bioengineering Graduate Program, American University of Sharjah, Sharjah P.O. Box 26666, United Arab Emirates; g00101100@aus.edu; 2 Department of Electrical Engineering, American University of Sharjah, Sharjah P.O. Box 26666, United Arab Emirates; khanm@aus.edu (M.N.A.K.); hnashash@aus.edu (H.A.-N.)

**Keywords:** anxiety, depression, electrical stimulation, hybrid EEG-fNIRS, mental health, psychological stress

## Abstract

Individuals experiencing high levels of stress face significant impacts on their overall well-being and quality of life. Electrical stimulation techniques have emerged as promising interventions to address mental stress, depression, and anxiety. This systematic review investigates the impact of different electrical stimulation approaches on these types of disorders. The review synthesizes data from 30 studies, revealing promising findings and identifying several research gaps and challenges. The results indicate that electrical stimulation has the potential to alleviate symptoms of anxiety, depression, and tension, although the degree of efficacy varies among different patient populations and modalities. Nevertheless, the findings also underscore the necessity of standardized protocols and additional research to ascertain the most effective treatment parameters. There is also a need for integrated methodologies that combine hybrid EEG-fNIRS techniques with stress induction paradigms, the exploration of alternative stimulation modalities beyond tDCS, and the investigation of the combined effects of stimulation on stress. Despite these challenges, the growing body of evidence underscores the potential of electrical stimulation as a valuable tool to manage mental stress, depression, and anxiety, paving the way for future advancements in this field.

## 1. Introduction

In accordance with the World Health Organization (WHO), psychological disorders are among the leading causes of morbidity, and an individual’s quality of life can be negatively impacted by such disorders [1]. In the year 2019, one in every eight individuals, which is equivalent to 970 million people globally, was living with a psychological disorder, with depression and anxiety in particular, constituting the most prevalent categories [2]. In 2021, 16% of young people encountered a significant episode of depression, and more than 2.7 million suffered from severe major depression [3]. According to the World Health Organization, there were 301 million individuals suffering from an anxiety condition in 2019, including 58 million children and adolescents [2]. Frequent psychological discomfort is associated with mental health disorders, long-term diseases, impairments in functioning, poor lifestyle choices, and high dependence on health services. Stigma, discrimination, and infringement of human rights are all endured by a significant number of individuals [4]. Research on the mental health of university students reveals that depressive symptoms are more widespread in this group than in the general population [1]. Levecque et al. (2017) reported that 51% of PhD students had a minimum of two mental health indicators, 40% experienced at least three, and 32% reported more than four symptoms. It was consistently observed that PhD students reported a greater number of symptoms than the general highly skilled population, staff members, and other students in universities [5]. Research indicates that 75% of mental problems appear before the age of 24 years, hence contributing to the heightened susceptibility of college students [6,7]. Stress, anxiety, and depression are prevalent mental health concerns among students, and they have the potential to adversely impact their holistic well-being involving physical, mental, and social aspects [1]. The tremendous progress in brain sciences and engineering has resulted in the swift emergence of novel and efficacious therapies for psychosocial and psychiatric illnesses. However, compared to other illnesses, creating medications for treatments involving the central nervous system (CNS) is more laborious, expensive, and unlikely to be effective. With the withdrawal of large pharmaceutical corporations from CNS drug research, alternative possibilities such as brain stimulation techniques are emerging [8]. Psychiatry has utilized brain stimulation for almost seven decades, first with electroconvulsive treatment (ECT) in 1938, which has subsequently become safer as a result of developments in anesthetics. Transcranial magnetic stimulation (TMS) was originally employed in 1985 as a non-invasive alternative to ECT for the treatment of illnesses such as depression and anxiety [8,9,10]. Brain stimulation generates action potentials without receptor binding, avoiding drug metabolism and adverse effects using contrast psychopharmacology [9].

The real-time, non-invasive assessment of physiological reactions during therapeutic treatments has great potential. This advancement is by the combination of wearable sensors combined with electrical stimulation methods. Recent developments in flexible and wearable electrode materials have improved the accuracy and convenience of healthcare monitoring, permitting tailored treatment techniques for mental health illnesses [11,12]. There is a substantial knowledge gap regarding the variability in treatment outcomes across various patient populations and stimulation modalities, despite the growing curiosity about electrical stimulation as an effective therapy for mental stress. In addition, the broader applicability of these interventions is restricted by the absence of defined protocols and extensive studies on alternative stimulation modalities. The systematic review is motivated by the need to explore the intricate connection between electrical stimulation and mental well-being, focusing on stress, depression, and anxiety and assessing the efficacy of a variety of electrical stimulation techniques in the management of psychological distress. This review aims to accomplish the following objectives:

- Assess the efficacy of a variety of electrical stimulation techniques.
- Provide an exhaustive overview of the current state of electrical stimulation research in mental stress.
- Identify critical challenges and research gaps in the field.
- Guide future developments in the field by achieving these objectives.

The various acronyms used throughout this review are summarized in Table 1.

## 2. Methodology

### 2.1. Inclusion Criteria

The Preferred Reporting Items for Systematic Reviews and Analysis (PRISMA) technique was utilized to conduct database searches for this review paper, which centered on English-language publications. To ensure comprehensive coverage, article retrieval was prioritized from primary databases, which were ScienceDirect, IEEE Xplore, and Scopus. Google Scholar and PubMed were utilized as supplementary resources to verify the information. Prominent journals, including those published by IEEE, Elsevier, Frontiers, and MDPI, were given due consideration. To supplement the existing literature, the review additionally incorporated secondary sources, such as conference papers and book chapters. The publication selection process included studies that examined the effects of electrical stimulation in subjects diagnosed with depressive or anxious disorders, as well as research designs that utilized electrical stimulation to mitigate stress-induced stress. Furthermore, the inclusion criteria included articles that utilized methods such as transcranial direct current stimulation (tDCS), transcranial alternating current stimulation (tACS), Functional Near-Infrared Spectroscopy (fNIRS), cortisol, and salivary alpha-amylase (sAA) to assess stress, depression, or anxiety and the effect of electrical stimulation. The inclusion requirements also included particular keywords pertinent to stress and electrical stimulation. Electroencephalography (EEG), fNIRS, and hybrid EEG-fNIRS were among the keywords utilized in the literature search.

### 2.2. Search Strategy

**Figure 1:**
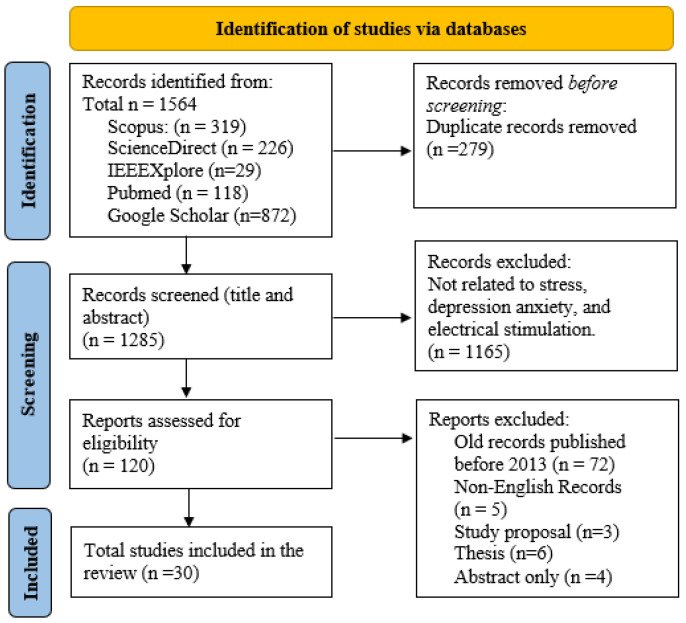
PRISMA flowchart outlining the search approach used for identifying suitable studies on mental health and tES.

The search strategy for this review, outlined in Figure 1, utilized an exhaustive method to identify research studies on stress, depression, anxiety, and the impacts of electrical stimulation. The search terms used were “stress”, “depression”, “anxiety”, and “electrical stimulation”, with additional refinements such as “stress AND measurement” and “mitigation AND cortisol AND alpha-amylase” to narrow down the results. Further specificity was obtained by incorporating terms such as “tACS” or “tDCS”. Shortened keyword combinations (such as EEG AND stress) were also utilized to improve efficiency. Prior to screening abstracts and titles for relevance, any duplicate entries were eliminated. Articles published prior to 2013, publications in languages other than English, and those that did not contain relevant keywords were not included in the analysis. Further studies were cross-referenced to identify relevant information. The analysis and results of this review are based on 30 carefully selected manuscripts that directly address stress, depression, anxiety, and the effects of electrical stimulation. In total, this paper includes 135 references. This broader reference list is crucial to guarantee an extensive foundation and context for the search. The additional references include basic theories, scientific guidelines, and important publications that offer a strong theoretical foundation for the review. In addition, they incorporate crucial research on relevant subjects, historical viewpoints, and recent breakthroughs that contribute to a broad awareness of the field.

Articles published prior to 2013, publications in languages other than English, and those that do not contain relevant keywords were not included in the analysis. Further studies were cross-referenced to identify relevant information. The analysis and results of this review are based on 30 carefully selected manuscripts that directly address stress, depression, anxiety, and the effects of electrical stimulation. In total, this paper includes 135 references. This broader reference list is crucial to guarantee an extensive foundation and context for the search. The additional references include basic theories, scientific guidelines, and important publications that offer a strong theoretical foundation for the review. In addition, they incorporate crucial research on relevant subjects, historical viewpoints, and recent breakthroughs that contribute to a broad awareness of the field.

## 3. Mental Stress, Depression, and Anxiety

Mental stress, affecting nearly two-thirds of the general population, is associated with a lower quality of life and health problems such as depression, diabetes, cancer, cardiovascular disorders, and stroke [24]. Stress can be acute or chronic, influenced by individual perceptions and coping abilities. Acute stress arises from specific events such as bereavement or loss of employment, while chronic stressors are more prolonged [25,26,27,28]. Stress activates the sympathetic–adrenal–medullary system and the hypothalamic–pituitary–adrenal axis, triggering physiological responses that prepare the body for a fight-or-flight reaction, including increased heart rate and energy mobilization [29]. Depression is a common and severe psychological condition linked to stress, often triggered by even minor stressors [30,31]. Stress and depression are closely related [32,33,34,35], and studies in rodents have shown that mild chronic stress can induce behaviors analogous to human depression, such as reduced motivation and altered sleep patterns [34,36]. Stressful experiences often trigger depression [37]. Anxious depression, a variant of major depressive disorder, involves comorbid anxiety in 40–50% of patients and shows poorer responses to antidepressants, leading to more severe symptoms and adverse effects [38]. Generalized anxiety disorder (GAD) is notably significant, with more than 70% of GAD patients experiencing severe depressive episodes [39]. The brain networks governing stress and anxiety are closely linked, suggesting that changes in these connections may contribute to conditions such as GAD, post-traumatic stress disorder (PTSD), and social anxiety disorders. Stress is a well-researched precursor of anxiety disorders, highlighting a bidirectional relationship between anxiety and stressful events [40,41].

## 4. Electrical Stimulation Techniques

**Figure 2:**
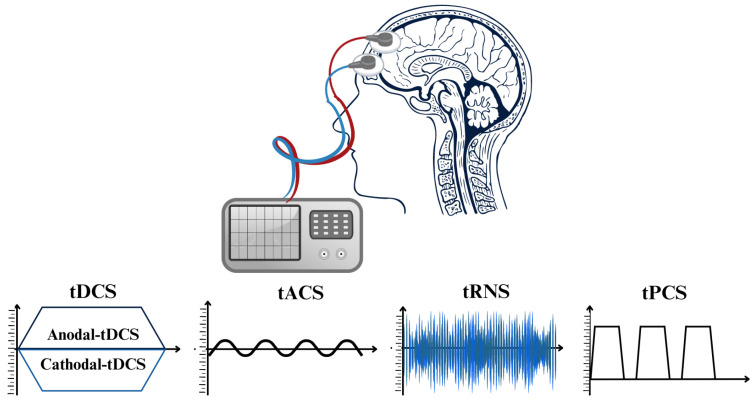
The diverse techniques of transcranial electrical stimulation (tES) procedures classified according to the unique waveforms of electrical current employed for stimulation.

Research and clinical practice have been significantly transformed through the implementation of non-invasive brain stimulation (NIBS) methods [42,43]. These techniques effectively regulate brain activity, thereby facilitating the comprehension of brain–behavior associations and the formulation of therapies for neurological and neuropsychiatric conditions. NIBS can induce long-lasting alterations in cortical stimulation and activation and alter neuronal activity during application [43,44]. Brain stimulation, under its phasic nature, induces immediate clearance, in contrast to pharmacological agents which require metabolism and elimination [9]. TMS and transcranial electric stimulation (tES) are modern NIBS techniques that exhibit dissimilar impacts on neurons. TMS produces powerful and brief electromagnetic currents in the cerebral cortex, resulting in a strong activation of the neurons over the threshold. On the other hand, tES does not cause neurons to produce action potentials. Instead, it influences their spontaneous firing activity by making small changes to their resting membrane potentials [43,45,46,47,48,49]. Depending on the stimulation parameters, both methods result in long-lasting neuroplastic alterations in neural pathways. Nevertheless, the simultaneous use of repetitive TMS and behavioral tasks has more challenges than tES, as excessive activations may interfere with task-related activity [43,50,51,52,53]. Nevertheless, tES is a valuable instrument in neurological study for exploring causal associations in neural activity, considering it is more cost effective, much simpler to use, and better adapted for double-blind studies as opposed to TMS, regardless of the aforementioned disparities [43,54]. Critical factors governing tES methodologies comprise electrode montages, current density, duration of application, and current intensity [55]. Multiple parameters associated with each tES method stimulate neuroplasticity through distinct mechanisms, thereby exerting various consequences on cortical neurons [56]. tES techniques can be classified into four primary subtypes according to the electrical currents in their waveforms (refer to Figure 2): tDCS [57], tACS [58], transcranial random noise stimulation (tRNS) [59], and transcranial pulsed current stimulation (tPCS) [56] are distinguished by their capacity to induce long-term neuroplastic changes through mechanisms such as spike-timing-dependent plasticity (STDP). In the subsequent sections, we investigate the four types of tES and their corresponding properties, which include electrode characteristics, current and frequency settings, dosage, ramp time, and the implementation of sham conditions.

## 5. Transcranial Direct Current Stimulation

tDCS is a non-surgical approach to stimulating the brain by delivering a low-level direct electrical current to the scalp utilizing electrodes [57]. It is a method that alters the polarity of neuronal membrane potentials and impacts the natural firing rate of neurons in the targeted area. The device utilizes a consistent current strength for stimulation, with gradual increases (ramp up) and decreases (ramp down) to reduce abrupt skin and ocular experiences. The current not only flows beneath the electrodes but also extends to the surrounding area and penetrates the brain tissue between them [59]. During stimulation, tDCS primarily produces two physiological effects: aftereffects mimicking long-term potentiation (LTP- and LTD-like) and the low-level modulation of membrane potentials. tDCS can cause changes in excitability that persist for more than 1 h after a brief application [59,60]. Conventional configurations frequently employ a two-electrode montage to ensure that the electric current has entry and exit sites on the scalp during tACS. The anode (positive electrode), as well as cathode (negative electrode), positions alternate continuously due to the fluctuating polarity of the current, facilitating the efficient modulation of neural oscillations throughout various regions of the brain [61].

The electrode assembly usually includes a metallic or conductive rubber electrode and a sponge pocket for electrodes, along with an electrolyte-based interface medium. An electrolyte should be used as an interface separating the electrode’s surface from the skin, preventing direct contact [57]. The electrolyte is typically housed in a sponge around the electrode or applied directly onto the surface of the electrode. However, when the electrode sponge is oversaturated, the repeatability of tDCS is compromised. This occurs because the saline spreads beyond the electrode surface area, resulting in inconsistent current delivery among different subjects. Ensuring that the electrode and scalp have proper contact, without being overly saturated, is crucial. It is important to note that any modifications to the size or assembly of the electrode can affect how current is distributed over the scalp and brain [57].

### 5.1. Parameter Optimization

#### 5.1.1. Electrode Size and Characteristics

Recent advances in electrode design for tDCS have emphasized the importance of using larger reference electrodes in conjunction with smaller, focal target electrodes to prevent substantial stimulation at the reference site. Studies have shown that the optimal electrode dimensions typically fall within the range of 25 to 35 cm^2^, with high-definition tDCS (HD-tDCS) employing specialized configurations such as ring electrodes composed of multiple smaller electrodes. These configurations enhance spatial focal point and circumvent issues associated with traditional electrode shapes, such as square sponges. However, while smaller electrodes improve focality, they may also increase skin irritation, which can be mitigated by adjusting the distance between electrodes [62,63,64].

#### 5.1.2. Electrode Location

In bihemispheric setups, where cathodal as well as anodal currents control opposite hemispheres differentially, precise electrode positioning is essential for effective stimulation [65]. The reference electrode is frequently positioned opposite to the target electrode in optimal configurations [66]. Current shunting by cerebrospinal fluid, which reduces cortical activation, is a risk associated with close electrode spacing [67]. Conversely, a wider spacing may be necessary to enhance cortical modulation, but it may also necessitate higher intensities, which may result in energy distribution [49,68,69].

#### 5.1.3. Current and Dosage

Optimizing the duration and intensity of tDCS to obtain the desired effects while maintaining safety is a critical aspect of research on tDCS. Several studies have indicated that stimulation periods ranging from five to thirty minutes and current levels ranging from one to two milliamperes are often investigated, with a suggested safety level of two milliamperes. Cortical excitability may be affected over an extended period of time by sustained stimulation, and the polarity effect is affected by the length of the stimulation [53,65,69].

The recommended dosage duration for tDCS stimulation is thirty minutes in order to reduce the likelihood of adverse side effects. Changes in excitability can be induced by stimulation lasting as little as four seconds, with prolonged sessions lasting seven minutes to provide more enduring effects [70]. Twenty-minute treatments per day are generally considered adequate, and newer models of tDCS devices for usage at home have safety safeguards to guard against abuse and guarantee mental health [70]. Furthermore, it is advised to gradually increase and decrease the intensity of the current in order to reduce skin burning, early sensitivity, and maintain an even distribution of current [70].

#### 5.1.4. Sham Conditions

Sham tDCS, often used in tDCS research, mimics skin sensations without altering brain excitability. It involves three stages: ramping up current, a brief stimulation, and ramping down. While effective for new users, it is less so for experienced ones [65].

## 6. Transcranial Alternating Current Stimulation

Transcranial alternating current stimulation (tACS) is a technique developed to investigate the cause-and-effect connection between brain oscillations and cognitive processes. It additionally has the potential to be used as a therapeutic tool for correcting disrupted oscillations in neurological disorders [71,72,73,74,75]. Administering an electrical current to the brain causes its endogenous oscillations to synchronize with the rhythm of the applied current, a phenomenon known as entrainment. This synchronization is the key concept of tACS. Neuronal oscillations, or periodic fluctuations in EEG activity across the brain, are to be modulated in order to enhance a variety of mental, subjective, and behavioral functions [61]. A frequency-dependent sinusoidal current is applied across the cranium; however, this method does not adhere to the polarity constraint that is observed in tDCS. The effectiveness of tACS is primarily influenced by the frequency, intensity, and phase of the treatment. The impact of tACS varies according to its intensity, with certain indications indicating that inhibitory networks are more responsive to lower stimulation intensities compared to excitatory networks. When tACS is delivered in the EEG range during human trials, it is predicted to primarily synchronize neural networks and perhaps improve information transit and accelerate processing. Repeated alteration of the synapse, when subjected to an oscillating electrical field, can also change related biochemical systems [67,76,77,78,79,80].

### 6.1. Parameter Optimization

#### 6.1.1. Electrode Properties and Current/Frequency Settings

Rubber or metallic electrodes having a skin–electrolyte interface area of 25 to 35 cm^2^ are commonly utilized in tACS. The effectiveness in administering the electrical current to the targeted brain areas relies heavily on these electrodes [61].

The magnitude of the alternating current utilized in tACS typically ranges from 0.5 to 2 mA. The range of the administered current frequency is varied between 0.5 and 80 Hz, contingent upon the particular neural oscillations that are being targeted. The amplitude is determined by comparing the peaks of the cycle’s positive and negative phases [61].

#### 6.1.2. Dosage and Ramp Time

In conscious humans, the minimum effective dosage (MED) for tACS is 0.23 mV/mm, as demonstrated by the meta-analysis performed by Alekseichuk et al. (2022). This value is substantially lower compared to the 0.49 mV/mm recommended to stimulate brain slices and anesthetized rodents. The active brain necessitates a lower dose, as the 80% probability of evoking a neural response in conscious mammals is achieved at 0.31 mV/mm as opposed to 0.67 mV/mm in anesthetized contexts. These results indicate that neural activity can be effectively modulated by sub-millivolt per millimeter electrical fields, which are feasible in humans at 2–4 mA. Furthermore, research has indicated that the effectiveness of tACS can differ based on different areas of the brain, the inherent neural activity, and the frequencies of stimulation [81].

#### 6.1.3. Sham Conditions

In tACS sham conditions, subjects undergo 30 s of tACS, which includes a 10 s fade-in and fade-out, at the onset of the stimulation period while keeping all other parameters unchanged [82].

## 7. Transcranial Random Noise Stimulation

Low-intensity tRNS is a non-invasive brain stimulation technique, where both the frequency and intensity of the electrical currents vary randomly. Initially developed to disrupt aberrant cortical oscillations, tRNS is now being explored for its potential to utilize stochastic resonance and other mechanisms. The stimulation is biphasic and can be administered with different noise types, typically following a Gaussian distribution centered around zero with a standard deviation, ensuring that 99% of current levels stay within ±1 mA. Notably, tRNS has shown effects similar to anodal tDCS and can enhance cognitive and brain functions sustainably [59,83,84].

Extensive studies have demonstrated that tRNS can increase excitability in particular cortical regions, which may result in long-term neurological and cognitive advantages. tRNS has the potential to affect brain blood flow, which could potentially lead to changes in neural connections [59,84,85,86,87]. Recent research has shown that the application of tRNS to the frontal lobes over a period of five days has been found to enhance arithmetic learning speed. This suggests that there is an improvement in neural and blood flow connectivity in the left dorsolateral prefrontal cortex (DLPFC) [88].

Increased sodium (Na^+^) channel opening rate and decreased gamma-aminobutyric acid (GABA) levels are two effects of tRNS that lead to increased cortical excitability. Functional imaging suggests that the effects of tRNS may be influenced by blood supply. High-frequency tRNS has been found to enhance visuomotor learning, whereas low-frequency tRNS may have a negative impact on it. From a scientific perspective, tRNS indicates to increase the excitability of neurons by aiding Na+ channels and decreasing GABA inhibition, which could potentially impact plasticity similar to long-term potentiation. There is evidence to suggest that tRNS could enhance neural signals in the presence of white noise, leading to potential improvements in sensory detection accuracy [89,90,91].

### 7.1. Parameter Optimization

#### 7.1.1. Electrode Charecteristics

The current density of tRNS is determined by the electrode size, which is essential for maintaining it within the safety limits of less than 1 A/m^2^, similar to tDCS. Saline-soaked sponges are frequently employed to cover large rubber electrodes, and safety precautions are necessary until particular guidelines for tRNS are defined [91].

#### 7.1.2. Current/Frequency

tRNS appears as white noise and operates within the frequency range of 0.1 to 640 Hz. Safety considerations guarantee that tRNS is reversible, non-invasive, painless, and marginally perceptible, thereby emphasizing its appropriateness for clinical and research applications [91].

#### 7.1.3. Dosage and Ramp Time

The intensity of tRNS is progressively increased in the initial 30 s and decreases in the final 30 s of stimulation, with a typical delivery time of 10–20 min. Critical parameters for the safe and effective administration of a dosage involve its duration and intensity [91].

#### 7.1.4. Sham Conditions

The sham condition in tRNS studies generally involves the application of current with a 30 s ramp-up and a 30 s ramp-down with no actual current after the ramp time [91].

## 8. Transcranial Pulsed Current Stimulation

tPCS is a non-invasive method of stimulating the brain that modifies neuronal activity by using surface electrodes placed on the auricular regions to provide an oscillating current to the brain [92]. This technology demonstrates favorable characteristics as a neuromodulatory tool since it is straightforward, devoid of discomfort, easy to operate, portable, and safe [93,94]. However, there is limited knowledge of its process of altering behaviors [94,95].

### 8.1. Parameter Optimization

#### 8.1.1. Electrode Properties and Current/Frequency Settings

Most tPCS electrodes have a size of approximately 16 square centimeters [94]. Previous research has commonly employed a range of intensities, frequencies, and stimulation durations in their studies. There have been several research studies that have investigated higher intensity and frequency ranges, such as 5–7 mA with frequencies of 7–11 kHz throughout 18–30 sessions. In animal studies, it has been observed that lower currents, such as 0.2 mA, are frequently used, along with frequency ranges of 10 Hz and 30 Hz. On the other hand, 0.1 to 0.35 mA intensities at frequencies as small as 2 Hz have also been employed [56]. According to Thibaut et al. (2017), studies showed that a tPCS current of 2 mA during a 20 min period of a random pulsing frequency between 6 and 10 Hz caused the most significant changes in brain activity. The findings show that a single session of tPCS is sufficient to elicit consistent alterations in the alpha bands of the brain (global, low, and high alpha) when the random frequencies parameter is set to between 6 and 10 Hz [96].

#### 8.1.2. Dosage and Ramp Time

The session durations reported in most studies ranged from 6 to 20 min. Certain studies have explored the effects of prolonging the stimulation duration to 30 min. When tPCS is combined with transcutaneous electrical nerve stimulation, treatments could last up to 30 min over the course of 60 sessions [56].

#### 8.1.3. Sham Conditions

The sham tPCS is administered with same current strength and oscillation frequency. However, it is only supplied for a duration of 30 s, after which the device will disconnect immediately [93].

## 9. Biomarkers and Imaging Modalities

An effective method for investigating neurocircuitry in healthy subjects as well as those with neurological conditions is to combine neurophysiology, functional imaging, and brain stimulation. In order to help develop novel therapies, this approach permits the monitoring of intermediate results. It is feasible to perform imaging or physiological assessments simultaneously with stimulation procedures [9].

### 9.1. Biomarkers

The response of the body to stress, whether it arises from mental, physical, or emotional factors, involves a variety of biological and psychological alterations. The autonomic nerve system (ANS) is responsible for regulating the activities of internal organs and ensuring that the body remains in a state of balance. External factors, such as stress, have the potential to disrupt the functioning of the ANS, resulting in various physiological effects that vary depending on the individual’s perceived stress levels [97].

#### 9.1.1. Cortisol

Cortisol serves as the principal hormone that carries out the effects of the hypothalamic–pituitary–adrenal (HPA) axis stress response mechanism. The HPA axis, similar to other components of the endocrinal system, is controlled by a negative feedback mechanism [98]. This means that the hypothalamus and pituitary gland possess receptors that sense fluctuations in cortisol levels [99,100]. When cortisol levels increase, the secretion of cortisol is suppressed, and when cortisol levels decrease, its secretion is stimulated. Repeated activation of the HPA axis leads to an increase in cortisol production, resulting in excessive levels of the hormone in body tissues [26,97,100]. Continued activation of the body’s systems due to repetition can cause harm to tissues and contribute to future health problems [27,29]. Sachar’s study on hypercortisolaemia in depression highlights the notable cortisol response in individuals with elevated levels of plasma cortisol at rest [37]. Mineralocorticoid receptors (MRs) and glucocorticoid receptors (GRs) are the two types of corticosteroid receptors that are found in the brain [37]. Cortisol binds to both MRs and GRs, with MRs exhibiting a 10-fold higher receptor affinity for cortisol [37]. MRs are responsible for controlling the baseline HPA activity while you are under normal conditions [37]. However, when you are experiencing stress, GRs become increasingly engaged, which indicates that the HPA axis activity is decreased. The activation of MRs and GRs enables the nervous system to react effectively to varying levels of cortisol [37,101,102].

#### 9.1.2. Alpha Amylase

sAA is widely recognized as an accurate predictor of autonomic nervous system activity. Its secretion can be affected through both parasympathetic and sympathetic stimulation. Protein concentrations such as sAA tend to increase with sympathetic activation, while fluid secretion is enhanced by parasympathetic activity. Both autonomic branches have the potential to increase sAA levels, particularly when they are stimulated together. This enzyme, which is primarily generated by the parotid and submandibular glands, is essential for the initial digestion and binding to oral microbes. Krirschbaum et al. (1993) noted that in order to confirm sAA as a stress indicator, further research is required, with a focus on studies that utilize standardized stress tests [101,103].

#### 9.1.3. Blood Pressure

High blood pressure is an avoidable cause of mortality that has a substantial impact on the quality of life of patients. Acute and chronic stress is believed to play a role in the onset of hypertension. Acute stress induces the “fight or flight” response, which results in an increase in heart rate as well as blood pressure. Conversely, chronic stress can result in persistent elevated arterial hypertension and other cardiovascular risks. The prolonged activation of chronic stress can lead to a range of health problems, highlighting the significance of stress management in reducing the risk of cardiovascular disease [104].

#### 9.1.4. Heart Rate Variability

Heart rate variability (HRV) is a measure of the variation in time between successive heartbeats. It provides insights into the functional state of the ANS [105,106,107]. It captures the equilibrium between both parasympathetic and sympathetic activity, with low HRV suggesting increased sympathetic drive and possible pathological conditions. The advantages of HRV include its straightforwardness, cost effectiveness, mobility, non-intrusiveness, and ease of analysis. However, variations among individuals and the absence of standardization can affect the outcomes [105,106,107]. In comparison to other stress indicators like cortisol and catecholamines, HRV provides a more immediate and sensitive measure of stress. This helps in monitoring stress habituation and preventing chronic health issues as noted by [105,106,107].

### 9.2. Imaging Modalities

Cognitive functioning is dependent upon complex neurophysiological mechanisms that comprise alterations in cerebral hemodynamics, brain electrical activity, and ANS function throughout the execution of cognitive tasks. fNIRS, EEG, and HRV can individually (unimodally) or concurrently (multimodally) measure these changes [105]. EEG is a technique that researchers commonly use to measure the electrical activity of neurons in the brain’s cortex. It offers valuable insights into neurophysiological processes and provides excellent temporal resolution [105]. Utilizing cutting-edge EEG techniques, such as brain mapping, connectivity analysis, and event-related potentials (ERPs), significantly improves the precision of diagnoses and cognitive research [105,108,109]. EEG allows for the study of brain communication by analyzing functional and effective connectivity. It can also map active cortical areas while performing tasks. Unlike behavioral assessments, EEG provides objective and quantitative neurophysiological data, which lend a more scientific perspective [105,108,109]. fNIRS is a valuable tool in studying cortical hemodynamic changes through neurovascular interactions. It offers benefits such as portability and improved temporal resolution. However, it is important to note that it has limitations in terms of spatial resolution and penetration depth [105,110,111,112]. One notable advantage of employing the multimodal approach is that it effectively addresses the drawbacks associated with single modality measurements. There is a growing body of research that prioritizes the concurrent application of non-invasive modalities, such as combined fNIRS-EEG, EEG-HRV, and fNIRS-HRV, to improve performance and information. Research has shown that classification accuracy can be enhanced by concurrently employing multiple measurement modalities [105]. To date, however, and as far as our knowledge extends, no published study has investigate the effects of electrical stimulation on anxiety, depression, or stress using the previously mentioned multimodal approach.

## 10. Results

**Figure 3:**
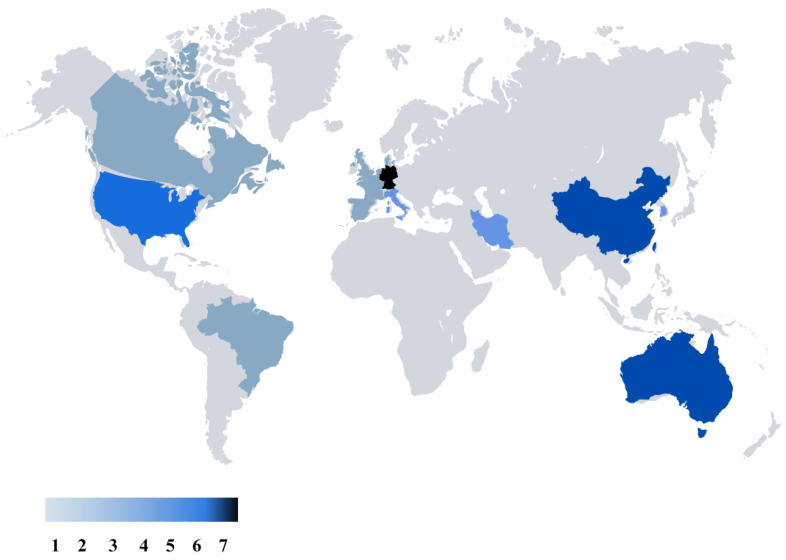
Worldwide distribution of research on transcranial electrical stimulation for stress, depression, and anxiety, emphasizing major contributing regions and research volume.

Figures that were created after our literature review are presented in this part as the results. Figure 3 represents the global map of the nations that have conducted studies related to electrical stimulation for the mitigation of depression, stress, or anxiety. Germany is in the lead with seven publications, while Australia and China are tied closely behind with six publications each. There are five studies from USA, and four studies from Korea. These numbers underscore a global interest and effort in exploring the potential of TES in addressing stress, depression, and anxiety, reflecting a diverse landscape of research and clinical initiatives worldwide.

**Figure 4:**
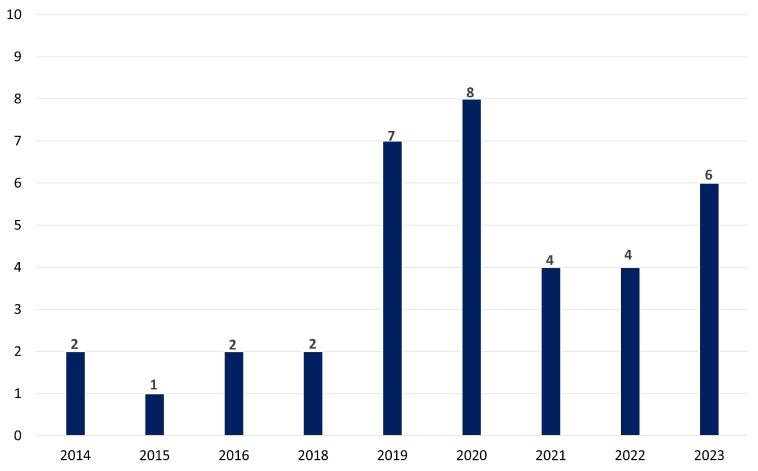
The temporal patterns in tES research throughout the last decade emphasizing the increasing awareness of tES as a viable therapy for mental health challenges.

Figure 4 represents the temporal distribution of the selected papers for the impact of electrical stimulation on stress, depression, or anxiety in the last decade. Over a decade, from 2014 to 2023, the research interest in exploring the potential of tES as a tool for addressing stress, depression, and anxiety appears robust. The tally of publications reflects this trend, with a total of 30 articles dedicated to investigating tES in the context of mental health concerns. The distribution of these publications shows a steady increase over the years, with peaks in 2019 and 2020, suggesting a growing recognition of tES as a promising intervention in this field. These findings also underscore the ongoing efforts within the scientific community to uncover innovative approaches to managing and treating psychological distress, leveraging the potential of neuromodulation techniques like tES.

**Figure 5:**
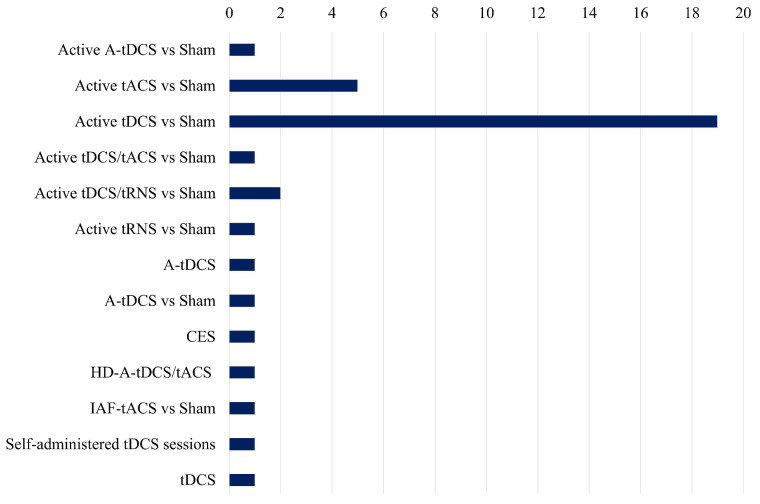
Comparative study of several tES methodologies employed in research, highlighting their prevalence and emphasis in current studies.

Figure 5 presents an unbiased comparison of the frequency of different electrical stimulation methods utilized in the studies analyzed in the review paper. Active tDCS vs. Sham is the most extensively researched method, with almost twenty references, indicating its dominant position in present-day research settings. This implies that the primary focus of investigators has been on the potential therapeutic effects of tDCS on stress and related disorders.

There is a gap in the literature for other types of electrical stimulation like tACS, while approaches like active tACS vs. Sham are investigated significantly less frequently. The under-representation of combined approaches, such as tDCS/tACS and tDCS/tRNS, in comparison to tDCS alone, suggests that there is a necessity for additional investigation into the synergistic effects of various stimulation methods. Furthermore, the duration of stimulation sessions differs among studies, but it typically falls within the range of 12 to 30 min, with current intensities spanning from 1 to 2 mA. Although some studies concentrate on healthy control populations, others investigate clinical populations, emphasizing the potential of electrical stimulation interventions across a variety of groups. Nevertheless, there are significant research voids, such as the dearth of exhaustive investigations into the combined effects of stimulation and stress and the limited exploration of alternative stimulation modalities beyond tDCS. Additionally, the necessity of standardized methodologies and longitudinal designs to improve the generalizability and reproducibility of findings is readily apparent.

### 10.1. Mental Stress

Antal et al. (2014) [13] conducted a study with a sample size of 60 healthy controls (HC) comparing active versus sham transcranial direct current stimulation (tDCS) at Fz- for cathodal and Fp2/O2-P4 for anodal stimulation (refer to Table 1). The sessions lasted for 20 min over three days, with a current intensity of 1 mA. The task involved the Trier Social Stress Test (TSST), and measurements included cortisol levels and functional magnetic resonance imaging (fMRI). Positive effects were observed for both anodal and cathodal tDCS. Similarly, Mario Bogdanov et al. (2016) [14] recruited 120 HC and compared anodal, cathodal, or sham tDCS with different montages: A-tDCS F4/Cz and C-tDCS with reversed polarity. Their sessions lasted 30 min with a current intensity of 1.075 mA, including an 8 s fade-in and 5 s fade-out. They assessed cognitive and emotional tasks like cognitive behavior therapy (CBT) and DSB-VM alongside physiological measures like the TSST, blood pressure, pulse, and cortisol levels, finding positive effects for anodal tDCS. Austin et al. (2016) [15] examined 66 HC in a study comparing active versus sham tDCS with a montage of F3/F4, lasting 12 min with a current intensity of 1.5 mA. They reported positive effects, particularly regarding premenstrual syndrome. Carnevali et al. (2020) [16] explored 30 HC using anodal tDCS versus sham with electrodes placed at F3/F4 for 15 min with a current intensity of 2 mA. Their tasks involved stress interviews and arithmetic tasks, with measurements including heart rate variability, TSST, CES-D, and cortisol levels, showing positive effects. Ankri et al. (2020) [17] investigated 69 HC comparing tDCS versus sham with a montage of F4/Cz for 20 min with a current intensity of 2 mA. Their task involved the N-back test, with measurements including TSST, State-Trait Anxiety Inventory (STAI), cortisol, and visual analog scale, yielding inconclusive results. Kim et al. (2020) [18] examined 60 HC comparing single-session tDCS versus gamma-tACS with a montage of F3/F4 for 30 min. They reported positive effects on resting-state EEG. Brunelin et al. (2021) [19] studied 30 HC comparing active tDCS versus sham with a montage of F3/F4 for 20 min with a current intensity of 2 mA, observing positive effects on cortisol levels. Ghafoor et al. (2022) [20] investigated 15 HC using anodal high-definition tDCS (A-HD-tDCS)/tACS over the prefrontal cortex (PFC) for 12 min with a current intensity of 1 mA for tACS. Their measurements with fNIRS showed positive effects. Wandel et al. (2023) [21] recruited 60 HC comparing tDCS versus sham with a montage of F3/FP2 for 20 min with a current intensity of 2 mA, finding no effects. Lee et al. (2023) [22] examined 62 HC comparing cranial electrotherapy stimulation (CES) versus sham on both temples for 30 min during the day and night for three weeks. Their measurements included various psychological scales and cortisol levels, showing positive effects. Lastly, Liu et al. (2023) [23] explored 40 HC comparing anodal tDCS versus sham with a montage of F3/F4 for 20 min daily over five days with a current intensity of 2 mA. They reported positive effects on attention networks, perceived stress, state anxiety, and EEG measurements.

Although tDCS has potential for alleviating the symptoms associated with stress, the wide range of study designs, stimulation settings, and outcomes underscores the necessity for more research. Standardized approaches and long-term investigations are necessary to enhance our comprehension of its effectiveness and build dependable, therapeutically valuable regimens for stress treatment using electrical stimulation techniques.

### 10.2. Depression

The research conducted by various authors on tES techniques, including tDCS, tACS, and tRNS, aimed to investigate their efficacy in treating major depressive disorder (MDD) (refer to Table 2). Powell et al. (2014) [113] explored the effects of tDCS on visual working memory tasks among 18 MDD patients, finding positive effects after active stimulation. Similarly, Loo et al. (2018) [114] studied 130 MDD patients, observing positive effects on depressive symptoms following active tDCS. Alonzoa et al. (2019) [115] reported positive effects on depressive symptoms among 34 MDD patients who self-administered tDCS. These studies collectively suggest the potential of tDCS as a promising intervention for MDD. Contrastingly, Alexander et al. (2019) [116] found no significant effects of tACS on depressive symptoms among 10 MDD patients. However, Nishida et al. (2019) [117] reported positive effects of anodal tDCS on both MDD patients and healthy controls, indicating potential benefits beyond clinical populations. Haller et al. (2020) [118] demonstrated the positive effects of active tACS on cognitive tasks among MDD patients. Conversely, Nikolin et al. (2020) [119] reported the negative effects of tRNS on cognitive function and mood in MDD patients. Huang et al. (2021) [120] found positive effects of tDCS on depressive symptoms and functional magnetic resonance imaging (fMRI) markers among MDD patients. Arabi et al. (2022) [121] observed positive effects of tDCS on EEG activity among MDD patients. However, Huang et al. (2024) [122] reported negative effects of tDCS on depressive symptoms among MDD patients when targeting specific brain regions. Pedraz-Petrozzi et al. (2023) [123] found negative effects of tDCS on cortisol levels among MDD patients, suggesting potential physiological side effects. Similarly, Murphy et al. (2023) [124] found no significant effects of various tES techniques on cognitive tasks among MDD patients. At present, there is only one report that pertains to the management of major depressive disorder (MDD) and the impact of tRNS on mood. A patient with major depressive disorder who had previously responded to two trials of tDCS (2 mA, 20 min each session, 15 sessions over three weeks) before receiving a four-week course of open-label tRNS (2 mA with 1 mA direct electrical current offset, 20 sessions of 20 min each) was presented by Chan et al. (2012) [125]. There was a 63% decrease in the severity of symptoms associated with depression from baseline by the fifteenth tRNS session. This was a considerable improvement over the two previous tDCS studies, which had end-of-acute-treatment reductions of 31% and 25%, respectively. The patient’s baseline depression levels were comparable across all three trials; however, tRNS resulted in rapid recovery and less cutaneous sensation than tDCS. These promising results, along with the possible theoretical benefits of tRNS over tDCS, provide strong evidence that the antidepressant effects of tRNS merit more research. Therefore, the efficacy of tES techniques in treating MDD varies across studies, with tDCS showing promising results in alleviating depressive symptoms and improving cognitive function in some cases.

Overall, tES approaches, namely tDCS, have shown significant potential in reducing depressive symptoms and enhancing cognitive function in individuals diagnosed with major depressive disorder. However, the inconsistent results shown in different trials emphasize the necessity for more study. The varied outcomes, including favorable, impartial, and even adverse impacts, underscore the need for discerning the most advantageous stimulation parameters, personalized therapy methods, and underlying processes. Furthermore, the growing possibility of tRNS, supported by notable advancements in certain instances, indicates that this approach deserves additional exploration to gain a deeper understanding of its therapeutic advantages in relation to other methods like as tDCS and tACS.

### 10.3. Anxiety

In a comprehensive review of studies on tES and its impact on GAD, several key findings emerged from the research conducted by various authors (refer to Table 3). Movahed et al. (2018) [129] conducted a study with 18 participants, comparing active tES against sham tDCS, with electrodes placed at the left deltoid and F4 locations. The sessions lasted 20 min each, conducted over a span of 10 sessions within 4 weeks, with a current intensity of 2 mA. Results indicated positive effects on anxiety levels as measured by the Hamilton Anxiety Rating Scale (HARS), Hamilton Depression Rating Scale (HDRS), and Penn State Worry Questionnaire (PSWQ). Similarly, Lin et al. (2019) [130] examined the effects of tES on GAD with a sample size of 20 participants. Their study, also comparing active tDCS against sham, involved electrodes placed at the left mastoid and F4 locations. The sessions lasted 20 min per day for 10 days, with a current intensity of 2 mA. Results showed significant positive effects on anxiety levels as measured by the Hamilton Anxiety Rating Scale (HAMA) and Hamilton Depression Scale (HAMD). Contrary to these findings, Lima et al. (2019) [131] explored the impact of tES on GAD with a sample size of 30 participants. Their study, comparing active tDCS against sham, positioned electrodes at the F3 and right FP2 locations. Sessions lasted 20 min each for 5 days, with a current intensity of 2 mA. Surprisingly, the results showed neutral effects on anxiety levels as measured by the HAM-A, Beck Anxiety Inventory (BAI), Impact of Event Scale (IES), Positive and Negative Affect Scale (PNAS), and Beck Depression Inventory (BDI). In another study focusing on the effects of TES, Mehrsafar et al. (2020) [132] conducted research with 12 healthy controls, examining the impact of anodal tDCS (A-tDCS) and cathode tDCS (C-tDCS) compared to sham tDCS. Electrodes were placed at the F3/F4 locations for A-tDCS and in reverse for C-tDCS. Sessions lasted 20 min each, with a current intensity of 2 mA. This study assessed the effects on performance in an official archery competition and physiological markers such as the Bowel Movement Stress Scale (BMSS), State-Trait Anxiety Inventory (STAI-II), and cortisol levels. The results indicated positive effects on performance and anxiety reduction.

Although tES, especially anodal stimulation, reduces anxiety in GAD, study findings vary, highlighting the need for additional research. These discrepancies arise from variations in electrode location, session duration, and task types. Future research should prioritize the development of standardized protocols and larger sample sizes in order to optimize tES for anxiety treatment.

## 11. Discussion

The global distribution of studies demonstrates significant interest, notably from Germany, Australia, and China, indicating a broad research landscape. The temporal analysis reveals a significant and steady rise in research interest throughout the last ten years, reaching its highest point in 2019 and 2020. This highlights the increasing acknowledgment of tES as a potential therapeutic. The tES techniques, such as tDCS, tACS, tRNS, and tPCS, present unique advantages and disadvantages that influence their application in both therapeutic and research contexts. tDCS has gained significant attention owing to its cost effectiveness, user-friendly application, and non-invasive characteristics, thereby facilitating its utilization in both clinical settings and home environments. Nonetheless, challenges emerge in identifying the most effective electrode positioning, defining stimulation parameters, and achieving reliable outcomes across different subjects. tACS provides enhanced precision in modulating brain activity via sinusoidal currents, which is advantageous for the exploration of cognitive functions. However, it is important to note that its effects may be impacted by the indirect stimulation of the peripheral nerves. It may also induce disruptions in visual processing within specific frequency ranges. Although tRNS and tDCS have comparable therapeutic potential, tRNS has the added benefit of being less susceptible to placebo effects in research. However, its processes and long-term consequences are little known. Lastly, although being relatively new and understudied in the field of stress research, tPCS has potential for further research, but there are currently few therapeutic uses for it. The limitations of each tES approach highlight the need for more study to enhance their clinical application [133,134,135].

This review of studies examining the effects of electrical stimulation on stress reveals a diverse landscape of findings. While several studies reported the positive effects of tDCS on stress-related outcomes, including cortisol levels and psychological measures, the efficacy varied across different montages, stimulation parameters, and tasks. Anodal tDCS appeared to be particularly promising in alleviating stress-related symptoms in healthy controls. However, inconsistencies were noted, with some studies yielding inconclusive results or showing no significant effects. Further research is warranted to elucidate the optimal stimulation protocols and target populations for maximizing the stress-relieving potential of electrical stimulation techniques. Additionally, future studies could benefit from standardizing methodologies and incorporating longitudinal designs to assess the long-term effects of electrical stimulation on stress management. Overall, while there is preliminary evidence supporting the beneficial effects of electrical stimulation on stress, more robust and well-controlled studies are needed to establish its clinical utility definitively.

The efficacy of tES techniques in treating MDD varies across studies, with tDCS showing promising results in alleviating depressive symptoms and improving cognitive function in some cases. However, inconsistencies in findings, such as negative effects observed in some studies, warrant further investigation into individualized treatment protocols, optimal stimulation parameters, and underlying mechanisms to maximize therapeutic outcomes and minimize adverse effects. However, while there is promising evidence suggesting the efficacy of tES, particularly anodal stimulation, in reducing anxiety levels among individuals with GAD, there are variations in outcomes across different studies. Factors such as electrode placement, session duration, and task outcomes may contribute to the differences observed in the results. Further research with larger sample sizes and standardized protocols is warranted to establish the optimal parameters for tES interventions in managing anxiety disorders.

**Figure 6:**
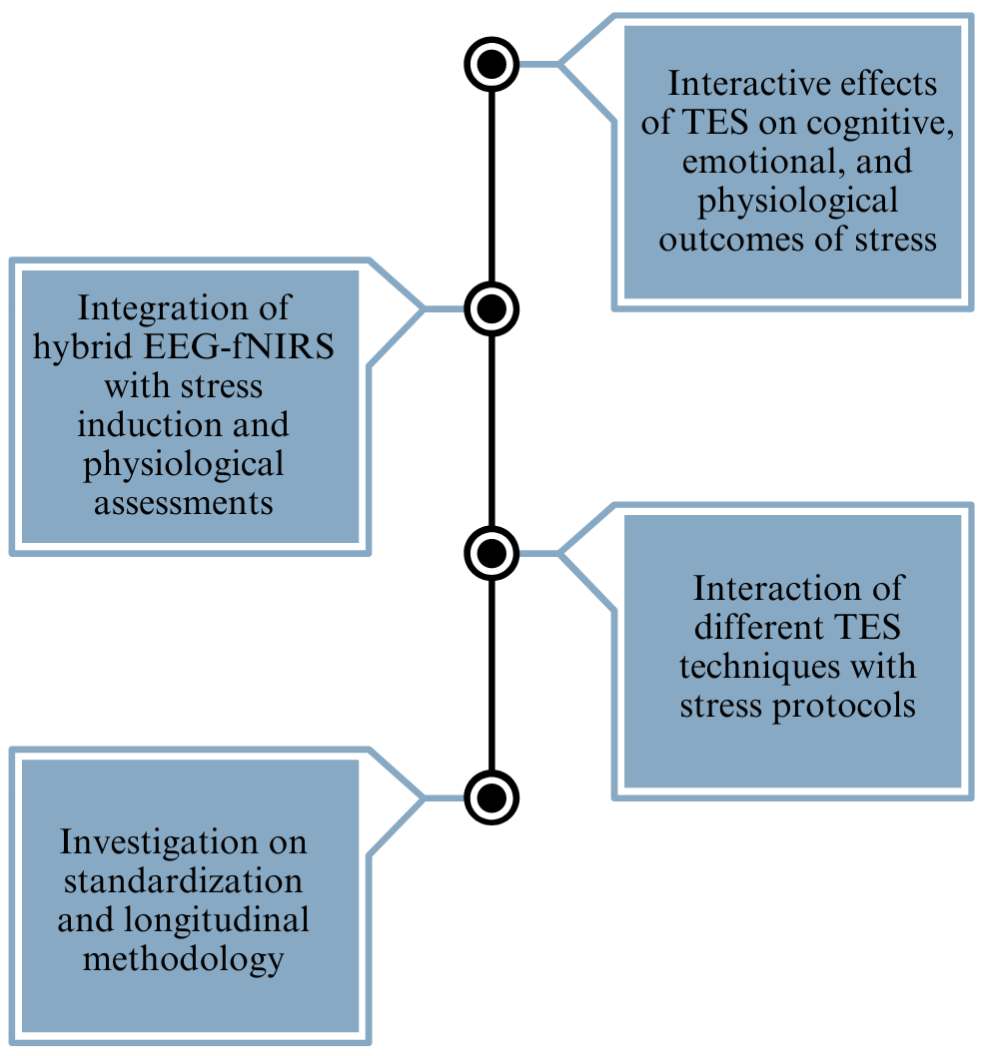
A brief overview of the identified research gaps, providing a clear picture of the primary challenges addressed in the study.

The reviewed studies provide valuable insights into the effects of electrical stimulation on mental stress, depression, and anxiety. Several research gaps and challenges persist in the field (refer to Figure 6). One notable gap is the absence of studies integrating hybrid EEG-fNIRS methodologies with stress induction paradigms and comprehensive physiological assessments, such as cortisol and alpha-amylase levels, within a single experiment. Combining these techniques could offer a more comprehensive understanding of the neurobiological mechanisms underlying stress responses and the modulation effects of electrical stimulation. Furthermore, there is a scarcity of research focusing specifically on the interaction between electrical stimulation techniques, such as tACS, tPCS, and tRNS, and stress induction protocols. While tDCS has received considerable attention in stress-related studies, exploring the potential of these alternative stimulation modalities in modulating stress responses could provide valuable insights into their comparative efficacy and mechanisms of action. Secondly, the current research heavily emphasizes the comparison between tDCS and a sham treatment, indicating the necessity for a more extensive investigation into alternative stimulation techniques, such as tACS and tRNS. The discrepancies in results among different studies, especially the lack of consistency in the impact on stress, depression, and anxiety, emphasize the need for standardized approaches and long-term research designs to improve the capacity to replicate and apply findings to a wider population. Thirdly, while some studies have examined the effects of electrical stimulation or stress induction protocols independently, there is a lack of comprehensive research exploring the interactive effects of stimulation and stress on cognitive, emotional, and physiological outcomes. Investigating how electrical stimulation influences stress resilience, coping strategies, and affective regulation under different stress conditions could provide valuable insights into the adaptive mechanisms of the brain and inform the development of targeted interventions for stress-related disorders. Finally, many studies in the field have employed cross-sectional designs with varying methodologies, making it challenging to compare results across studies and establish robust conclusions. Incorporating longitudinal designs and standardizing methodologies, such as stimulation parameters, stress induction protocols, and outcome measures, could enhance the reproducibility and generalizability of findings. Longitudinal studies would also enable researchers to investigate the long-term effects of electrical stimulation interventions on stress resilience and mental health outcomes.

Therefore, even though the reviewed studies offer promising evidence of the beneficial effects of electrical stimulation on mental stress, depression, and anxiety, there are notable gaps in the literature regarding methodological approaches, stimulation modalities, and target populations. Addressing these gaps through well-designed empirical studies could advance our understanding of the complex interplay between electrical stimulation and mental health outcomes, ultimately informing the development of more effective interventions for stress-related disorders.

## 12. Conclusions

Stress has emerged as a significant issue in today’s society. It disturbs an individual’s equilibrium, with the extent of its effect being contingent upon both the severity of the stressor as well as the individual’s capacity for resilience. This review highlights the efficacy of electrical stimulation techniques in mitigating symptoms associated with stress, depression, and anxiety. Despite the observation of promising results, the inconsistencies present across various studies underscore the necessity for standard procedures and the optimization of stimulation protocols. The literature reveals significant gaps, particularly in the exploration of alternative techniques such as tACS and tRNS. Additionally, there is a notable deficiency in comprehensive research addressing the interactive effects of current stimulation and stress. Future research should concentrate on improving these approaches, using longitudinal designs, and investigating hybrid neuroimaging techniques in order to gain a deeper understanding of the treatment processes. Overall, even though electrical stimulation appears promising, more thorough and systematic studies are required to prove its therapeutic value. The burgeoning field of electrical stimulation holds great promise for improving our ability to manage and alleviate the burden of mental stress, depression, and anxiety in society.

## Figures and Tables

**Table 1:** Transcranial electrical stimulation studies for mental stress.

Reference	Sample Size	tES Type	Session Duration	mA/Hz	Task	Outcomes Measurement	Results
[13]	60 healthy controls	Active vs. Sham tDCS	20 min over 3 days	1 mA	TSST	Cortisol fMRI	Positive A-tDCS and C-tDCS effects.
[14]	120 healthy controls	Anodal, Cathodal, or sham tDCS	30 min	1.075 mA	CBBT and DSB-VM	TSST, MMQ, BP, pulse, and cortisol	A-tDCS: Positive effects
[15]	66 healthy controls	Active vs. Sham tDCS	12 min	1.5 mA	NA	PMS	Positive effects
[16]	30 healthy controls	A-tDCS vs. sham	15 min	2 mA	Stress interview and arithmetic task	HRV, TSST, CES-D and Cortisol	Positive effects
[17]	69 healthy controls	tDCS vs. Sham	20 min	2 mA	N-Back	TSST, STAI-T, cortisol and VAS	Inconclusive
[18]	60 healthy controls	Single-session tDCS vs. gamma-tACS	30 min	tACS: 2 mA (40 Hz) tDCS: 2 mA	NA	Resting state EEG	Positive effects
[19]	30 healthy controls	Active tDCS vs. Sham	20 min	2 mA	Maastricht acute stress test	Cortisol	Positive effects
[20]	15 healthy controls	A-HD-tDCS/tACS	12 min	tACS: 1 mA (10 Hz) tDCS: 1 mA	NA	fNIRS	Positive effect
[21]	60 healthy controls	tDCS vs. Sham	20 min	2 mA	MIST	EFT, VAS, BSRI, and cortisol	No effects
[22]	62 healthy controls	CES vs. Sham	30 min during Day and 30 min during night for 3 weeks	10 Hz and 0.3 mA	NA	PSS, ISI, PSQI, BDI-II, STAI-S, WHOQOL-BREF, cortisol and QEEG	Positive effects
[23]	40 healthy controls	A-tDCS vs. sham	20 min for 5 days	2 mA	Attention Network Test	PSS, STAI, and EEG	Positive effects

**Table 2:** Transcranial electrical stimulation studies for depression.

Reference	Sample Size	tES Type	Session Duration	mA/Hz	Task	Outcomes Measurement	Results
[113]	18 major depressive disorder patients	Active vs. Sham tDCS	20 min	2 mA	Visual working memory task	MINI, MADRS, and EEG	Positive effects
[114]	130 major depressive disorder patients	Active vs. Sham tDCS	30 min	2.5 mA	NA	MADRS	Positive effects
[115]	34 major depressive disorder patients	Self administered 20–28 tDCS	30 min	2 mA	NA	QIDS-SR, MADRS, BDI and Q-LES-Q-SF	Positive effects
[116]	10 major depressive disorder patients	Active vs. Sham tACS	40 min	1 mA each/−2 mA (ref) 10 Hz/40 Hz	NA	HDRS, MADRS, BDI, and hdrEEG	No effects.
[117]	14 major depressive disorder patients and 19 healthy controls	Active vs. Sham A-tDCS	20 min	1 mA	STAI	Resting State EEG	Positive effects
[118]	6 major depressive disorder patients	Active tACS	10/20 min per day for 10 days	2 mA at 40 Hz (0 face shift)	3N-back task	HAMD21, BDI, RWT, TMT-A/B, PANAS and CGI	Positive effects
[126]	1 major depressive disorder patients	Active vs. Sham tACS	40 min	1 mA each/−2 mA (ref) at 10 Hz	NA	MADRS and hdrEEG	Positive effects
[127]	93 major depressive disorder patients	Active vs. Sham A-tDCS	30 min	2 mA	NA	HDRS and MODA	Positive effects
[119]	66 major depressive disorder patients	Active vs. Sham tRNS	30 min	2 mA and offset of 2 mA	Digit span subtest	MADRS, CGI-I, BDI-II, Q-LES-SF, CVLT-II, RUFF, WAIS-IV, SDMT and KEFS	Negative effects
[120]	112 major depressive disorder patients	Active vs. Sham tDCS	30 min per day for 10 times	2 mA	NA	MADRS, HAMA, QIDS-SR, CGI and fMRI	Positive effects
[121]	36 major depressive disorder patients	Active vs. Sham tDCS	20 min for 10 days	2 mA	NA	EEG	Positive effects
[128]	100 major depressive disorder patients	Active vs. Sham tACS	40 min for 20 sessions	15 mA at 7.5 Hz	NA	HDRS-17	Positive effects
[122]	70 major depressive disorder patientsD	Active vs. Sham (rOFC/IDLPFC) tDCS	20 min	2 mA	NA	HAMD17, MADRS, and QIDS-SR	Negative effects
[123]	40 major depressive disorder patients	Active vs. Sham tDCS	30 min	2 mA	NA	Cortisol	Negative effects
[124]	49 major depressive disorder patients	Active A-tDCS, HF-tRNS or Sham	22 min	tDCS: 1 mA tACS: 1 mA with 1 mA Dc offset (100–640 Hz)	Sternberg WM task and PASAT	WAIS-IV, STAI and TMS-EEG	No effects

**Table 3:** Summary of transcranial electrical stimulation (tES) studies for anxiety.

Reference	Sample Size	tES Type	Session Duration	Intensity	Task	Outcome Measures	Results
[129]	18 GAD patients	Active vs. Sham tDCS	20 min/session, 10 sessions over 4 weeks	2 mA	NA	HARS, HDRS, PSWQ	Positive effects
[130]	20 GAD patients	Active vs. Sham tDCS	20 min/day for 10 days	2 mA	NA	HAMA, HAMD	Positive effects
[131]	30 GAD patients	Active vs. Sham tDCS	20 min/day for 5 days	2 mA	NA	HAM-A, BAI, ISSL, PNAS, BDI	Neutral effects
[132]	12 healthy controls	A-tDCS/R C-tDCS vs. Sham tDCS	20 min	2 mA	Archery competition	BMSS, STAI-II, Cortisol	Positive effects

## Abbreviations

The following abbreviations are used in this manuscript:

ACC	Anterior Cingulate Cortex
ACTH	Adrenocorticotropic Hormone
ANS	Autonomic Nervous System
A-tDCS	Anodal Transcranial Direct Current Stimulation
BLA	Basolateral Amygdala
CGI	Clinical Global Impression
CRF	Corticotropin-Releasing Factor
CNS	Central Nervous System
CVLT-II	California Verbal Learning Test II
C-tDCS	Cathodal Transcranial Direct Current Stimulation
DBS	Deep Brain Stimulation
DLPFC	Dorsolateral Prefrontal Cortex
EEG	Electroencephalography
ECT	Electroconvulsive Therapy
fMRI	Functional Magnetic Resonance Imaging
fNIRS	Functional Near-Infrared Spectroscopy
GAD	Generalized Anxiety Disorder
HAM-A	Hamilton Anxiety Rating Scale
HAM-D	Hamilton Depression Rating Scale
HC	Healthy Control
HDRS	Hamilton Depression Rating Scale
HD-tDCS	High-Definition Transcranial Direct Current Stimulation
HPA	Hypothalamic–Pituitary–Adrenal
MADRS	Montgomery–Åsberg Depression Rating Scale
MDD	Major Depressive Disorder
MINI	Mini International Neuropsychiatric Interview
MODA	Mood Disorders Questionnaire
PTSD	Post-Traumatic Stress Disorder
QIDS-SR	Quick Inventory of Depressive Symptomatology—Self-Report
Q-LES-Q-SF	Quality of Life Enjoyment and Satisfaction Questionnaire—Short Form
rOFC	Rostral Orbitofrontal Cortex
SAM	Sympathetic Adrenal Medulla
sAA	Salivary Alpha-Amylase
SDMT	Symbol Digit Modalities Test
STAI	State-Trait Anxiety Inventory
STDP	Spike-Timing-Dependent Plasticity
TMS	Transcranial Magnetic Stimulation
tACS	Transcranial Alternating Current Stimulation
tDCS	Transcranial Direct Current Stimulation
tES	Transcranial Electrical Stimulation
tPCS	Transcranial Pulsed Current Stimulation
tRNS	Transcranial Random Noise Stimulation
VMPFC	Ventromedial Prefrontal Cortex
VNS	Vagus Nerve Stimulation
